# Differential Correlation Across Subpopulations of Single Cells in Subtypes of Acute Myeloid Leukemia

**DOI:** 10.3390/biotech15030063

**Published:** 2026-08-05

**Authors:** Reginald L. McGee, Jake Reed, Gregory K. Behbehani, Kevin R. Coombes

**Affiliations:** 1 Department of Mathematics and Statistics, Haverford College, Haverford, PA 19041, USA; 2 Huntsman Cancer Institute, University of Utah Health Academic Medical Center, Salt Lake City, UT 84112, USA; 3 Department of Hematology, The Ohio State University Wexner Medical Center, Columbus, OH 43210, USA; 4 Department of Biostatistics, Data Science, and Epidemiology, Georgia Cancer Center at Augusta University, Augusta, GA 30912, USA

**Keywords:** acute myeloid leukemia, differential correlation, mass cytometry, correlations in context

## Abstract

Mass cytometers can record 40–50 parameters per single cell for millions of cells in a sample. Many methods have been developed to cluster phenotypically similar cells within cytometry data, but there are fewer methods to visualize activity and interactions of pairs of proteins across these populations. We have developed a workflow for analyzing correlations associated with predefined populations. By clustering blood samples from acute myeloid leukemia (AML) patients and normal controls using an established algorithm, we obtained a minimum spanning tree of clusters of single cells. Using surface marker expression, we identified clusters on the tree that belonged to phenotypes of interest. Next, we computed correlations between pairs of proteins in each cluster. We developed a novel, coherent, probability-based statistic to test differences between vectors of correlation coefficients. By comparing all combinations of the normal controls under the statistic, we created an empirical distribution that could provide a conservative threshold of differential correlation. Using this empirically derived distribution to define significance, we compared pooled samples from AML subtypes and normal controls to detect differential correlations. Given the structure present within this cytometry dataset, we found it natural to consider correlations in this manner versus aggregating all data and computing a single correlation. Our approach has the advantage that we can localize the statistical measure to determine contributions from particular phenotypic populations. Differentially correlated pairs of proteins can be further explored as possible testable hypotheses by considering a population’s distribution of correlation coefficients or biaxially plotting protein expressions within individual cells in a given population.

## 1. Introduction

Single cell technologies provide promise for gaining new insights into genetic diseases such as cancer, but the increasing dimensionality of these datasets makes analysis more complicated and the determination of mechanisms that promote the disease less obvious. Mass cytometry records expression levels for tens of proteins on hundreds of thousands of individual cells in collected samples [1]. Moreover, there is additional structure to the data in that each cell has a phenotype, and the samples can be collected from individuals who may have different subtypes of a disease of interest. Investigating high-dimensional datasets such as these requires the development of new methodologies, particularly methods that consider the varying levels of structure inherent to the dataset.

Acute myeloid leukemia (AML) is a genetic disease marked by an accumulation of immature myeloid cells in the bone marrow and eventually within the peripheral blood. For individuals diagnosed with AML, the 5-year relative survival rate is 28.5% [2]. The survival rates differ across subtypes of AML and thus understanding genetic and molecular differences between subtypes is of the utmost importance.

Classifying a subtype of cancer requires consideration of factors such as cytogenetics, molecular mutations, and the maturation and phenotype of the cancerous cells. A complication in developing treatments for AML is the heterogeneity observed in these factors. The disease can afflict a variety of cell populations and these populations can play different roles in the diseases. Two known cell populations are leukemic stem cells (LSCs) known to contribute to the initialization and recurrence of the disease [3] and leukemic regenerating cells (LRCs) now known to promote disease recurrence after chemotherapy [4]. Thus, metrics for quantifying the activity of cell populations in samples from different disease types, samples from individuals receiving therapy, and samples from the normal population could be useful for clinicians.

Specific karyotypes and mutations are often associated with different blood cancer subtypes. One avenue for attempting to understand the functions of these mutations is considering how protein interactions within cancerous phenotypes differ from normally observed interactions. Historically, as sequencing data became more ubiquitous, new techniques for data analysis were necessary to determine co-regulated genes and to better understand prognostic factors [5,6]. It is natural to develop similar techniques as high-dimensional cytometry data continues to become more prominent. We seek to develop techniques for detecting differences in protein co-regulation across both patients and cellular phenotypes.

An advantage of using mass cytometry when studying cancers like AML is that it can capture biological phenomena at the level of single cells and is particularly suited to the analysis of complex cell populations. Gating strategies, the consideration and comparison of different parameters collected during a cytometry panel, can be used to identify phenotypes of the cells present in a sample and then to visualize them in two dimensional plots. The identification of phenotypes can be aided by recent techniques for analyzing cytometry data; these range from projections from higher-dimensional spaces, where the data lives, into two and three dimensions [7,8,9] and clustering algorithms that produce trees of clusters where the branches are phenotypically related [10,11,12,13]. In both cases, the expression of surface proteins one at a time can help to gate the projected or clustered data. Moreover, there has been work considering differential abundance of cell populations in mass cytometry data [14,15] given that determining the type of blood cancer and rate of progression depends on the phenotype most closely related to the malignant cells that have accumulated.

In addition to differential abundance of cell populations, differential expression of intracellular proteins is also of interest in cytometry research [1,16]. A visual analysis of one intracellular protein at a time can be conducted after the clustering or projection methods above have been applied. These approaches can give more prognostic insight into biological features within specific phenotypes, but do not provide information on co-regulation since they only consider one protein at a time. Thus, there is a need for tools that consider more than one protein at a time, akin to the expansion of co-expression analyses in microarrays [17]. Considering two proteins at a time in the context of cytometry has been considered via analyses of their conditional variation [18,19], but their correlation has yet to be considered. In the case of two proteins, determining co-regulation starts with finding positive correlations between the proteins. Given that multiple cell populations can be identified within mass cytometry data, co-regulation between two proteins could be determined either for an entire sample or for specific cell populations. Our approach analyzes pairs of proteins across samples that can also be localized to specific cell populations of interest within each sample. The advantage here is that the approach can be applied to other single cell technologies and, more generally, to any structured collection of correlation coefficients.

In this manuscript, we present a technique for determining when protein interactions change in aggregate between samples being compared. That is, we consider when there is a significant change in protein correlations within cells of the observed phenotypes between two samples being compared. To illustrate the approach we consider when there are significant differences between samples from patients diagnosed with distinct subtypes of AML as this could point towards hypotheses for underlying mechanisms driving the disease. We begin by describing the AML data used in this study and discuss how it was processed as well as the cell count variability across samples and across subtypes. Next we give a brief description of the established agglomerative clustering algorithm applied to all samples aggregated together, emphasizing that the surface marker expression from each sample was passed to the algorithm to produce a consistent minimum spanning tree of clusters of cells that are phenotypically similar. We then discuss how the clustered data was processed and begin the derivation of the statistic we use to test differential correlation between samples. In the Section 3, we have presented results of applying the derived statistic and use traditional gating techniques to investigate the significant changes in correlation detected for particular phenotypes. Finally, we discuss steps for discerning whether detected relationships can lead to impactful testable hypotheses.

## 2. Materials and Methods

### 2.1. Data Description

We investigate existing acute myeloid leukemia (AML) data for intracellular correlations that undergo statistically significant changes when comparing different subtypes of the disease.

The mass cytometry data, originally collected by Behbehani et al. [3], consisted of 60 protein readings for 58 samples in ten groups, with eight groups being different AML subtypes. The original study sought to determine aberrant protein expressions in the AML subtype samples when compared to normal controls. Aberrance was determined by a 2-fold difference outside the total variance of the normal samples one protein at a time.

The subtypes considered, as shown in Table 1, were: core-binding factor AML (5 samples), t(8;21) or inv(16) karyotype (4 samples), normal karyotype with a FLT3-ITD mutation (11 samples), normal karyotype with wildtype FLT3 (5), adverse risk karyotype (7 samples), and normal karyotype with FLT3-TKD mutation (4 samples). In addition to these groups, there was a group of fourteen normal control samples and a group of six samples that responded positively to chemotherapy. In all subsequent plots, the subgroup colors were chosen with the version 1.6.1 of the R package Polychrome [20].

**Figure 1:**
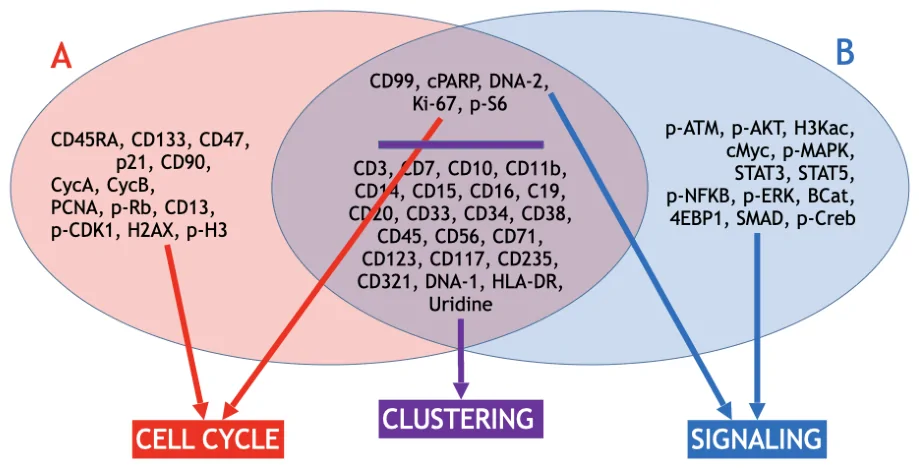
Summary of the proteins measured in the mass cytometry data collected in the study (Behbehani et al.) [3]. The red and blue ovals represent collections of proteins (panels) that were measured, with the two panels corresponding to either cell cycle (**tube A**) or intracellular signaling dynamics (**tube B**). There are common proteins (purple) between the panels; these are largely surface proteins used to cluster individual cells by their phenotype. Differential correlation analysis was performed with the proteins distinct to the cell cycle or cell signaling panel (red or blue, respectively).

There were 53 proteins measured that were split across two cytometry panels, each panel having a total of 47 proteins (Figure 1). There were 21 surface proteins as well as five intracellular proteins that were consistent across the two panels. Each panel had 13 unique intracellular proteins corresponding to cell cycle or signaling proteins. The other proteins were used for barcoding or were not appropriate to be considered in a correlation study. Thus, a total of 18 proteins will be used for our analysis.

### 2.2. Workflow

**Figure 2:**
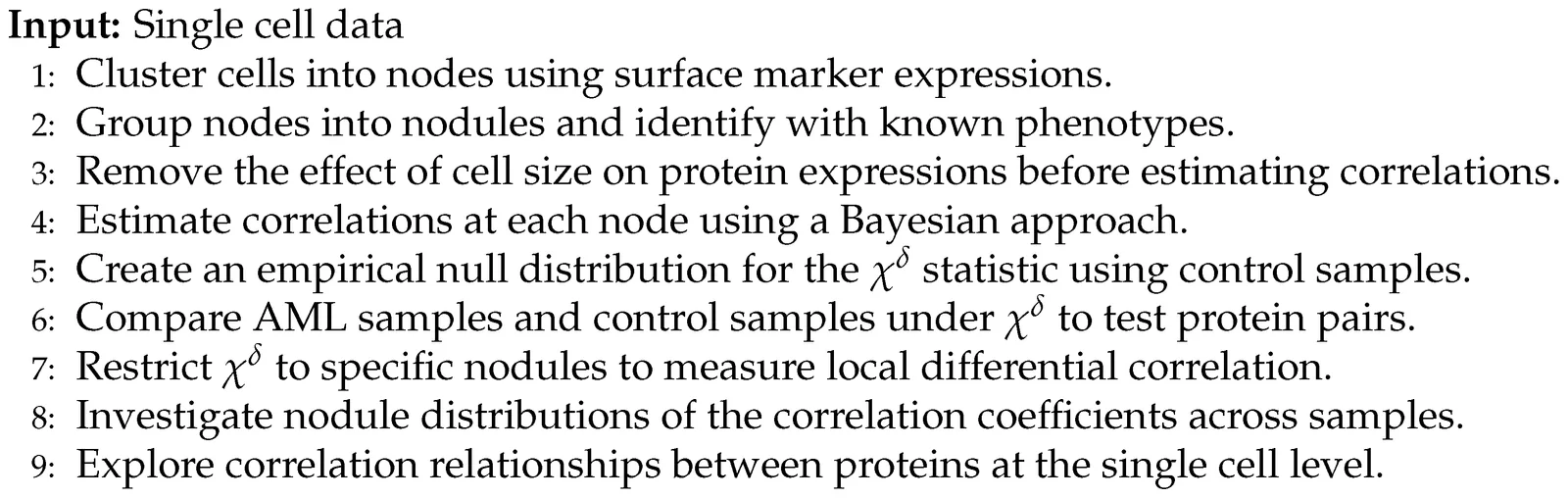
Workflow for identifying differentially correlated proteins pairs. The global differential correlation measure can be restricted to a particular nodule to arrive at a local measure of differential correlation. Investigation and exploration of local differential correlation in nodules of interest can be accomplished with bean and biaxial plots. Localization of the statistical measure helps to put the correlation relationships into specific contexts.

An overview of the workflow to identify differentially correlated vectors is shown in Figure 2. We note we will discuss a specific clustering algorithm next, but step 1 of the workflow is not restricted to a particular algorithm. We also note that the methods in steps 5–7 apply generally for any collection of correlation coefficients. Additionally, when subcollections of correlation coefficients have phenotypic meaning, the grouping in step 2 of the workflow can be accomplished manually or by a number of automated tools [12,13,21,22,23].

### 2.3. Clustering with SPADE

Spanning tree Progression of Density normalized Events (SPADE) [11] is an approach to extract information about hematopoietic development from mass cytometry data. SPADE uses agglomerative clustering and downsamples dense regions of cells before clustering to ensure rare cell types are not missed. After connecting the nodes into a minimum spanning tree the regions are upsampled and cells are placed into appropriate nodes.

The dataset we consider was clustered in R with SPADE version 1.18.2, using 19 of the 21 surface markers common in the two panels. During a previous analysis with this dataset [3], the surface marker CD56 was omitted because of the dimness of the reading and CD99 was used here during analysis as it has been previously used to correlate with disease. We sought a consistent minimum spanning tree for all patients and thus the 116 files were clustered together. In total, 483 nodes was the number of nodes specified to SPADE; the number 483 is chosen due to it being the square root of the average number of cells per sample [24].

**Figure 3:**
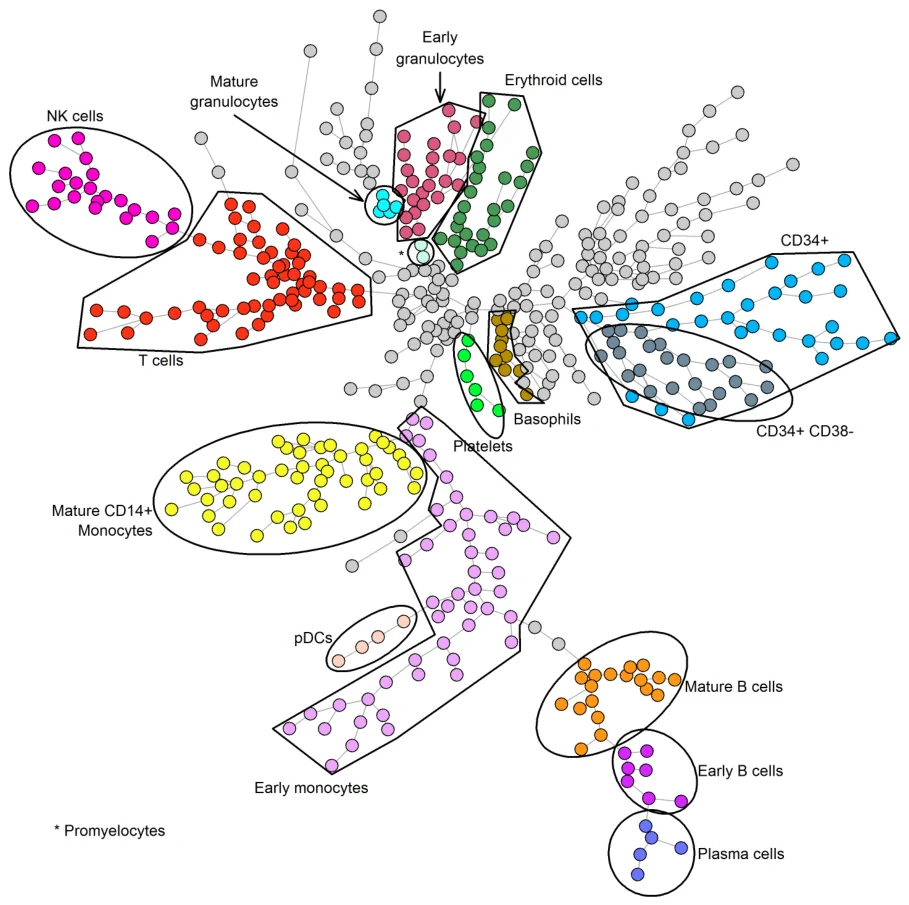
To ensure consistent comparisons between samples during our analysis, a single tree was created using surface marker expressions, shown in Figure 1, from all of the AML samples and samples from healthy donors. The tree was produced by Spanning tree Progression of Density normalized Events (SPADE) [11] considering all samples together; the nodes thus have consistent surface marker expression ranges across all samples. Nodes were reconciled with phenotypes using expressions from samples from healthy donors and this gives a sense of the cell phenotypes in the AML samples. There are 16 phenotypes annotated on the plot above and colored to be further distinguished. The remaining cells, left grey, could be classified, but the groups shown were those most of interest for this study.

More important than a precise number of nodes created by the algorithm are the phenotypes of the cells within those nodes. Minimum spanning trees produced by SPADE have the advantage that branches can be reconciled with known cell phenotypes. We define nodules as collections of nodes that can be identified with a phenotype. The nodules were determined manually by considering combinations of surface marker expressions, gates, from the samples from healthy donors, and then the normal cell phenotype groupings determined by the gates were projected onto the tree throughout the paper (Figure 3). Again, though in this study we used manual assignment of cells to phenotypes, the grouping step in Figure 2 could be accomplished through automated tools [12,13,21,22,23].

### 2.4. Processing Clustered Data

At each node in the minimum spanning tree, we estimated correlation matrices for the 18 proteins to be analyzed at each of the nodes through a Bayesian approach [25]. The estimation circumvents issues that arise when a node is empty or contains only a few cells and one attempts to correlate that data with built-in R functions. When there are no cells (N=0) in a given node, the method estimates the 18 × 18 correlation matrix $C_{N}$ for that node to be the identity matrix $I_{18}$. Assuming a Wishart prior distribution, the correlation matrix $C_{N}$ was estimated as follows when there are *N* cells in a node:  

$$\begin{array}{cc} a_{N} & =18+\frac{N}{2},\;\;\beta_{N}=1+N,\\ \bar{f} & =\frac{1}{N}\sum_{n=1}^{N}f_{n},\;\;\;\bar{\Sigma }=\frac{1}{N}\sum_{n=1}^{N}\left(f_{n}-\bar{f}\right){\left(f_{n}-\bar{f}\right)}^{T},\\ B_{N} & =I_{18}+\frac{N}{2}\left[{\bar{\Sigma }}_{N}+\frac{1}{\beta_{N}}\bar{f}{\bar{f}}^{T}\right],\\ C_{N} & =\frac{1}{a_{N}}B_{N}. \end{array}$$

Thus, when there are many cells in a node, the covariance estimate in $B_{N}$ then overwhelms the prior $I_{18}$. Correlation matrices are estimated at every node, and stored in the folders named according to that node, for individual samples, the all-sample pool, and each subtype pool. For instance, in the case of Core-binding factor AML, a subtype pool would mean at each node correlating protein expressions from cells for all five samples assigned to that node during clustering.

### 2.5. Identifying Statistically Significant Changes in Protein Pairs

In order to find statistically significant changes between the group of normal control samples and groups of samples with different subtypes of AML, we sought a distribution on the Pearson correlation coefficients and created a test statistic $\chi^{\delta }$ (Equation (1)) related to a Chi Square, $\chi^{2}$, statistic. Given a protein pair *x* and *y* of the 18 proteins in the panel reserved for analysis, there is a correlation coefficient at each of the 483 nodes for each group of samples. When comparing all possible pairs, this resulted in 153 vectors $P_{xy}^{\left(j\right)}=\left(\rho_{x_{1},y_{1}}^{\left(j\right)},\rho_{x_{2},y_{2}}^{\left(j\right)}...,\rho_{x_{483},y_{483}}^{\left(j\right)}\right)$, where *j* is an individual sample or a group pooled by sample subtype and each component is a correlation coefficient between proteins *x* and *y* in the $i^{th}$ node determined by SPADE.

Our null hypothesis was that the correlations at node *i* arise from the same distribution and thus their distribution parameters were equal. A Fisher z-transform $f\left(r\right)=\frac{1}{2}\ln\left(\frac{1+r}{1-r}\right)$ [26,27] was applied componentwise to $P_{xy}^{\left(j\right)}$ for each *j*. This transform maps the components from a probability distribution on [−1,1] to a normal distribution with mean $\frac{1}{2}\ln\left(\frac{1+\rho_{x_{i},y_{i}}^{*}}{1-\rho_{x_{i},y_{i}}^{*}}\right)$ and standard deviation $1/\sqrt{N_{i}^{\left(j\right)}-3}$, where $\rho_{x_{i},y_{i}}^{*}$ is the true correlation coefficient and $N_{i}^{\left(j\right)}$ is the number of cells used to estimate $\rho_{x_{i},y_{i}}^{\left(j\right)}$. When considering the sum of the 483 squared standardized normal variables

$$c_{x_{i},y_{i}}\left(j,k\right)={\left(\frac{f\left(\rho_{x_{i},y_{i}}^{\left(j\right)}\right)-f\left(\rho_{x_{i},y_{i}}^{\left(k\right)}\right)}{\sigma_{f\left(\rho_{x_{i},y_{i}}^{\left(j\right)}\right)-f\left(\rho_{x_{i},y_{i}}^{\left(k\right)}\right)}}\right)}^{2}$$

we determined that our statistic for significant aggregate differences in correlation should, in theory, arise from a $\chi^{2}$ distribution with 483 degrees of freedom, denoted $\chi_{483}^{2}$.

Due to the number of cells in certain nodes being in the thousands, this posed numerical challenges during standardizing due to dividing by $1/\sqrt{N_{i}^{\left(j\right)}-3}$. We regularized our statistic by adding an exchangeability factor $s_{0}$ to the standard deviations in $c_{x_{i},y_{i}}\left(j,k\right)$ at each node *i*; an idea borrowed from Tusher et al. [28]. The values of $s_{0}$, shown in Table 2, is uniform for all sample comparisons for either cell signaling or cell cycle data. Since a Chi Square distribution is uniquely determined by its mean, the values of $s_{0}$ were determined by minimizing, over the interval [0, 2], the absolute deviation between the expected value of $c_{x_{i},y_{i}}\left(j,k,s_{0}\right)$ for all i,j,k,x, and *y* and the expected value of $\chi_{1}^{2}$. The upper bound on the interval [0, 2] arose from the case when nodes are empty. The minimization was performed with the one-dimensional optimization function optimize in R.

Intuitively, the addition of the $s_{0}$ term serves to prevent oversensitivity at nodes with a large number of cells by preventing the denominator from becoming too small and makes the statistic more conservative overall. Finally, we define our test statistic to be

(1) $$\begin{array}{c} \chi^{\delta }\left(j,k\right)≜\sum_{i=1}^{483}{\left(\frac{f\left(\rho_{x_{i},y_{i}}^{\left(j\right)}\right)-f\left(\rho_{x_{i},y_{i}}^{\left(k\right)}\right)}{\sigma_{f\left(\rho_{x_{i},y_{i}}^{\left(j\right)}\right)-f\left(\rho_{x_{i},y_{i}}^{\left(k\right)}\right)}+s_{0}}\right)}^{2}. \end{array}$$

To proceed with hypothesis testing, we chose to define an empirical null distribution using the $\chi^{\delta }$ statistic to compare the fourteen normal samples. The motivating idea was that an empirical distribution defined from normal comparisons would provide a baseline for significant differences one could expect when comparing vectors of cytometric correlations and anything beyond this baseline difference could confidently be considered significant. More about the empirical null distribution can be found in Appendix A.2.

We performed an ad hoc approach for minimizing the false discovery rate (FDR). For each of the nominal *p*-values of 0.1, 0.05, 0.01, and 0.001, we compute the proportion of findings determined to be significant in search of a satisfactory rate of discovery. Using the *p*-values, we determine a significance cutoff from the empirical distribution defined by normals and perform the $\chi^{\delta }$ comparison between each group of interest and the normal controls. Any statistics values beyond that of the cutoff that was determined by the normals are determined to be significant. The total number of significant comparisons is then normalized by the total number of nodes multiplied by the number of groups minus one. The addition of the number of nodes to the denominator is to account for the large number of comparisons happening in the $\chi^{\delta }$ testings, similar to that of a Bonferroni correction. When considering the nominal *p*-value of 0.001, we found the FDR value of 0.117 and 0.029 to be satisfactory for the cell cycle and cell signaling datasets, respectively. A summary of the number of significant findings and FDR values at each nominal *p*-value can be seen in Table 3. In the subsequent analysis, we choose the level p=0.001 for significance.

## 3. Results

We began by using SPADE to produce a minimum spanning tree that incorporated information about the surface markers from all samples in both the cell cycle and signaling experiments (Figure 3). Using the surface marker expression from the fourteen normal control samples, sixteen immunophenotypes of interest were manually reconciled with the nodes determined by SPADE. This reconciliation creates our immunophenotype nodules that are annotated atop the minimum spanning trees. For the purposes of this analysis, we refer to the unannotated cells on the diagram as the nodule of “other cells”. Moreover, the $\chi^{\delta }$ statistic can now be restricted to nodes from a particular immunophenotype. For the rest of the paper, the global $\chi^{\delta }$ statistic considers all 483 nodes, and local $\chi^{\delta }$ statistics consider nodes corresponding to particular phenotype nodules. To investigate a differentially correlated pair locally with respect to an immunophenotype nodule, we provide both biaxial plots of marker expressions of individual cells and the distribution of correlation coefficients.

### 3.1. Adjusting for Cell Size

In general, larger cells are expected to have higher levels of most proteins. Thus, before the main analysis, we normalized the data to adjust for the effect of cell size, which was measured by an iridium-based cell (IBC) marker. Specifically, we removed the IBC marker’s contribution to the expression of each feature by performing a linear regression between that feature and the IBC marker. All further analyses were performed on the residuals from these linear regressions. We note that since performing our analysis, there are now automated tools for removing confounding from mass cytometry data based around linear modeling [29].

**Figure 4:**
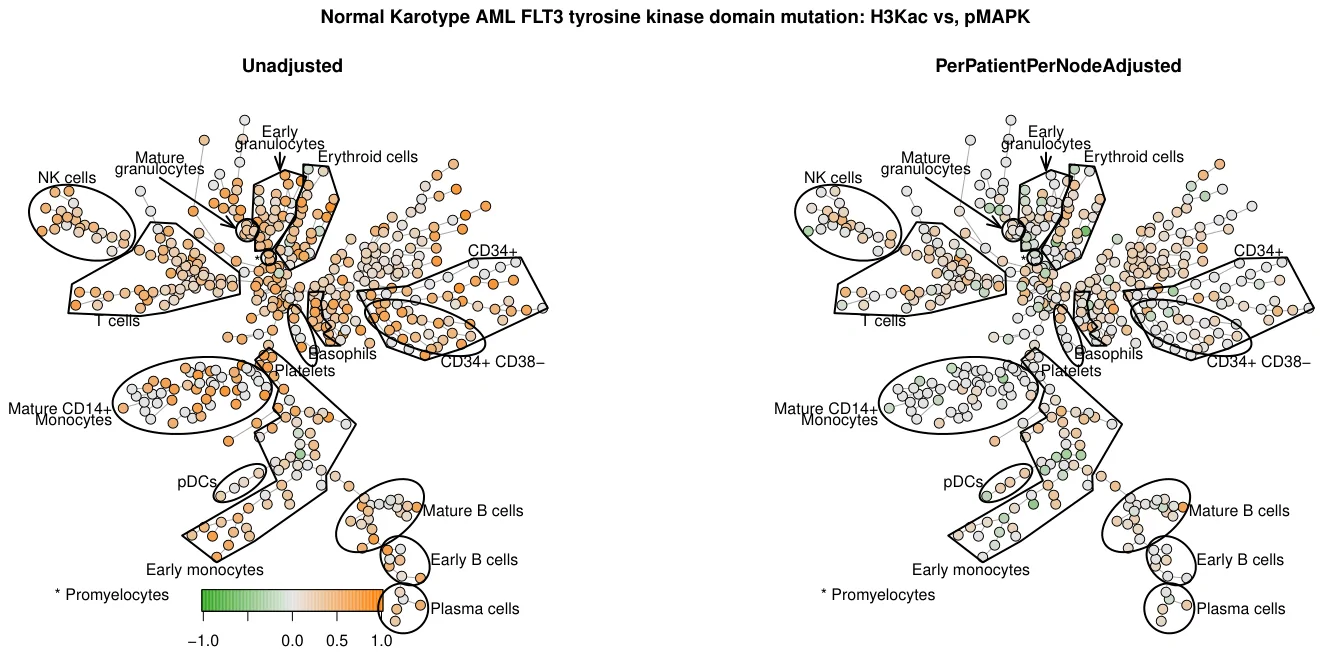
Illustration of how the adjustment for cell size at every node for each sample removes artificial positive correlation. The minimum spanning trees are colored by correlation coefficients between histone 3 lysine acetylation (H3Kac) and phosphorylated MAPK (pMAPK) on an absolute scale. The removal was motivated by the expectation that larger cells would have higher expressions of the measured proteins. On the left, the correlations are computed with unadjusted data and, on the right, the correlations are computed with residuals from the linear regression of each protein H3Kac and pMAPK on the contribution of the IBC marker (which measures cell size) for every sample at each node. Before the adjustment, the pair H3Kac and pMAPK were found to be globally differentially correlated. Overall, there is a reduction in positive correlation across the minimum spanning tree.

Shown in Figure 4 are minimum spanning trees before and after the cell-size adjustment for each sample at each node. These trees have been colored by the amount of correlation between a particular protein pair, histone 3 lysine acetylation (H3Kac) and phosphorylated mitogen-activated protein kinase (pMAPK), when comparing normal karyotype with FLT3-TKD mutation AML samples to normal controls. This pair of proteins was found to be differentially correlated before the cell-size adjustment but was not determined to be differentially correlated after adjustment. In total, 137 protein pairs were determined to be differentially correlated before adjusting for cell size but not after adjustment. The adjustment overall reduces correlation between the proteins and thus further increases the conservativeness of our results. For the rest of the paper, the data presented will have the per-sample-per-node cell-size adjustment.

### 3.2. Statistically Significant Changes in Correlation

**Figure 5:**
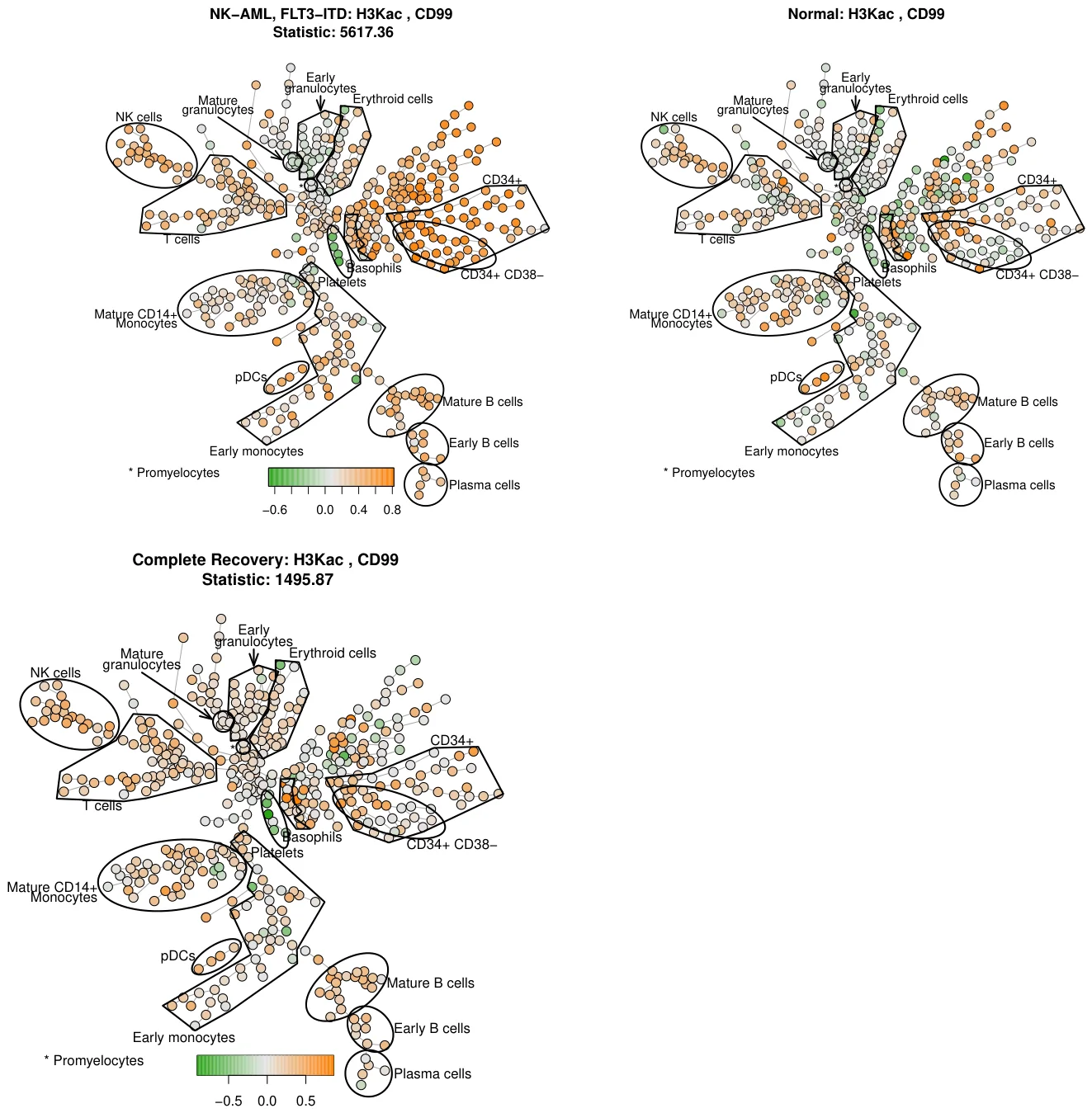
Minimum spanning trees colored by correlation coefficients between histone 3 lysine acetylation (H3Kac) and the CD99 antigen on a relative scale. Top: Correlations computed from pooled data from normal karyotype with a FLT3-ITD mutation (left) compared to pooled data from normal controls (right). Key visual differences can be seen in the CD34+ immunophenotype and CD34+ CD38− immunophenotype and in many other nodes. Bottom: Correlations computed from pooled data from patients in the group of patients without AML after chemotherapy. Key visual differences can be seen in many of the unannotated other nodes compared to the normals above, but less visual difference between the CD34+ CD38− immunophenotype. Overall there appears to be a decrease in correlation with the patients without AML after chemotherapy compared to the samples from healthy donors.

We computed $\chi^{\delta }$ statistics to test for changes in correlation vectors for each of the 153 possible protein pairs in each of the seven groupings of AML samples compared to normal control samples, leading to a total of 1071 comparisons. At the significance level p=0.001, using the cutoffs from the empirical null distribution (3046.27 and 1363.91, respectively), we found 37 significant pairs in cell cycle data and 150 significant pairs in cell signaling data. For both datasets, the normal karyotype with a FLT3-ITD mutation subtype had the most significant pair findings (cell cycle: 10; cell signaling: 35). Some of the differentially correlated pairs served as positive controls, some corresponded to known biological relationships, and some were potentially novel relationships that could be tested experimentally. In the subsequent analysis we will only report on the cell signaling data. The potentially novel protein pairs and the groups for which they were found to be differentially correlated compared to the samples from healthy donors are summarized in Table 4. How these global differential correlations results present locally, within the different cell phenotypes within the data, can be explored by plotting the correlation coefficients of the protein pairs atop the minimum spanning tree. We illustrate an example of this exploration in Figure 5.

### 3.3. Differential Correlation Between CD99 and H3Kac in CD34+ CD38− Cells

We illustrate the ability of our approach to generate testable hypotheses by considering the protein pair H3Kac and CD99. This pair was found to be differentially correlated for every group of AML samples when compared to normal control samples, but the immunophenotype nodules most contributing to the global $\chi^{\delta }$ statistic value varied drastically for each of the groups (Table 5). For this pair each group’s global statistic value was primarily driven by correlation at nodes from the other cells, but the variable contributions of other nodules highlight the advantage and necessity of considering local $\chi^{\delta }$ statistics.

To visually depict the variability in nodule contributions, we considered minimum spanning trees for the patients without AML after chemotherapy group and the normal karyotype with a FLT3-ITD mutation group versus the group of normal samples in Figure 5. For the CD34+ CD38− cell populations, we observe a strong visual change in normal karyotype with a FLT3-ITD mutation versus samples from healthy donors and a negligible change when comparing the patients without AML after chemotherapy versus samples from healthy donors. Quantified, the CD34+ CD38− nodule contribution (3.93%) for normal karyotype with a FLT3-ITD mutation is 2.5 times larger than that of the patients without AML after chemotherapy local $\chi^{\delta }$ statistic (1.57%).

**Figure 6:**
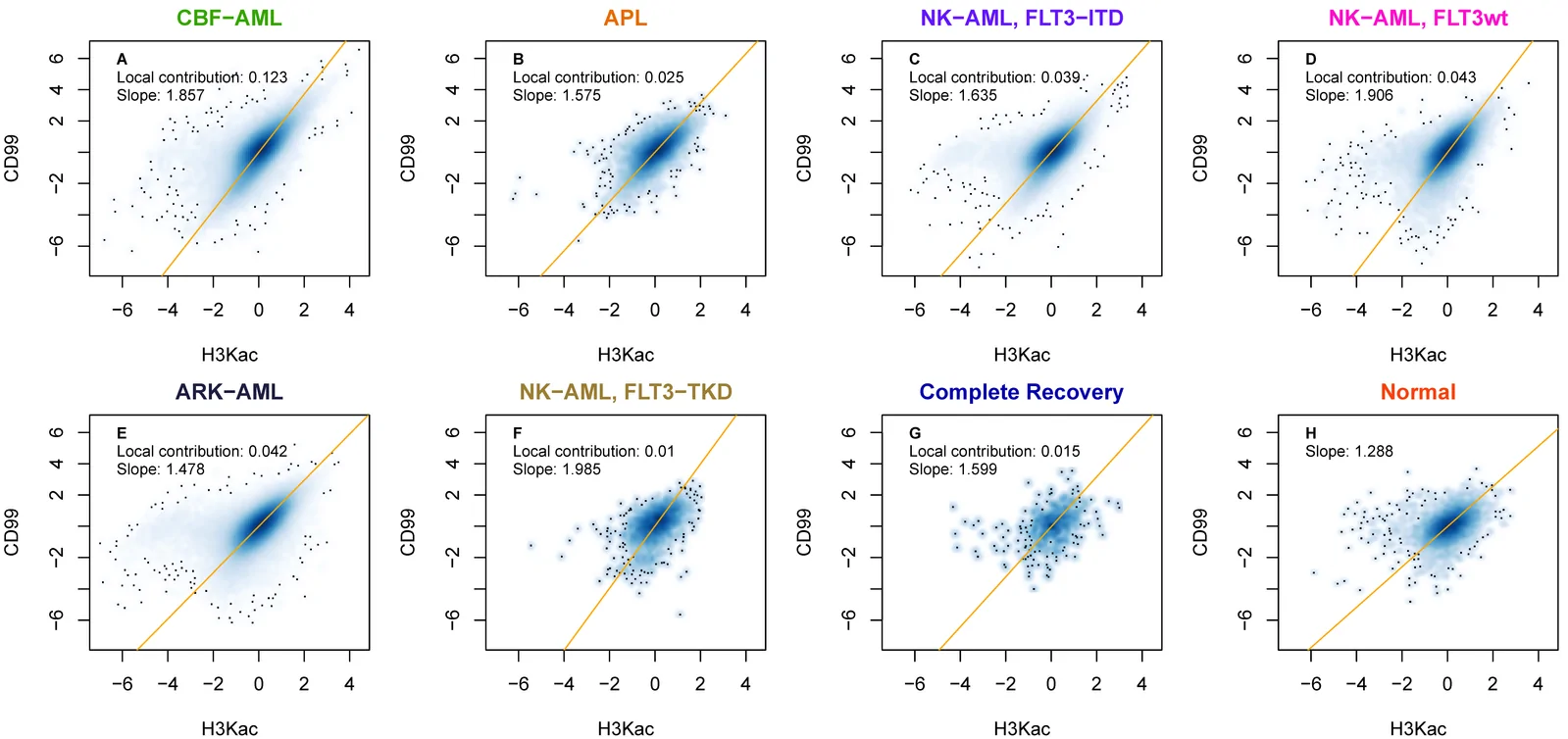
Biaxial plots of histone 3 lysine acetylation (H3Kac) expression versus CD99 antigen expression for individual cells in the CD34+ CD38− immunophenotype nodule. The local statistic contributions from Table 5 and slopes of the orange best fit line of the data cloud have been supplied. The protein pair was found to be globally differentially correlated for all sample group comparisons to the normal controls, but for this nodule strong positive correlation is only observed for the samples in Panels (**A**–**E**). The correlation is not seen in Panels (**F** or **G**), the sample groups for which the local statistic contribution are smallest, nor in Panel (**H**) for the normal controls.

When investigating whether a differential correlation finding is a worthwhile potential hypothesis, it may be advantageous to also consider whether the correlation is present in the raw data. The correlation between H3Kac and CD99 is present at the level of single cells for several groups when considering biaxial plots in Figure 6. A total regression line, which minimizes both horizontal and vertical displacements between data and the determined line, is added to each subplot in orange to show the overall correlation of the data cloud. The proteins are strongly correlated within the CD34+ CD38− cells, particularly in the case of core-binding factor AML, where the global statistic is driven to 21.48% by the other nodule and 12.31% by the CD34+ CD38− nodule. We note that the correlation is not present for patients without AML after chemotherapy nor samples from healthy donors.

**Figure 7:**
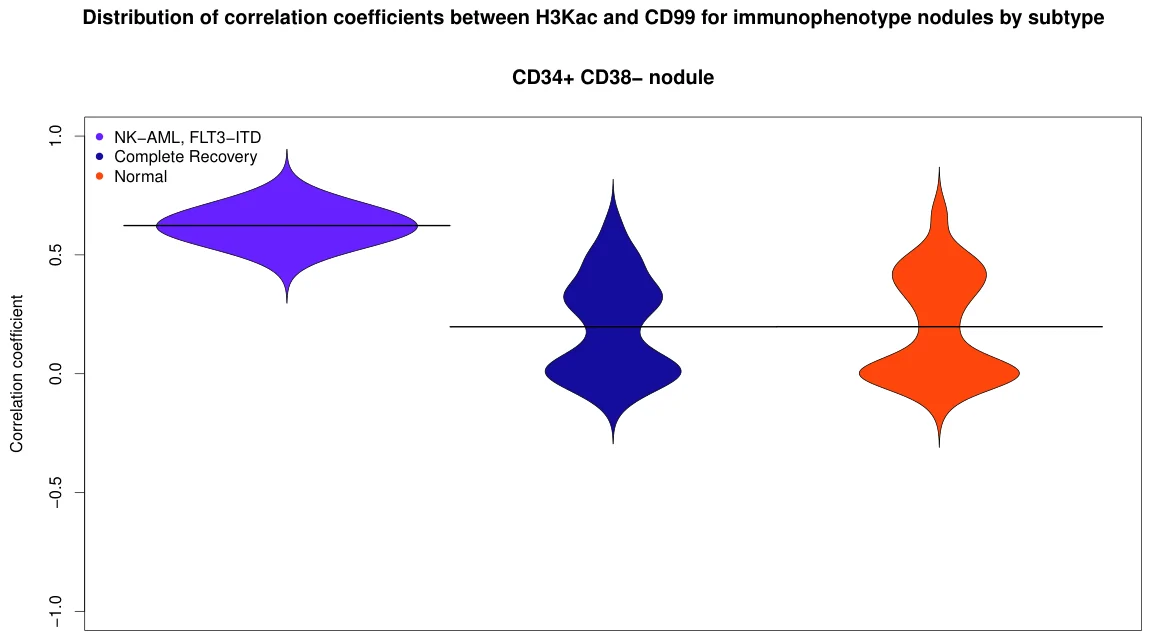
Bean plots depicting the distributions of correlation coefficients between histone 3 lysine acetylation (H3Kac) and the CD99 antigen at each node within the CD34+ CD38− nodule for three sample groups. The average correlation coefficient for each group is shown by the line through its beans. Comparing the distribution for the normal karyotype with a FLT3-ITD mutation sample to the distribution for the samples from healthy donors controls further illustrates the visual difference observed atop the minimum spanning trees in Figure 5.

To quantify visual differences in differential correlation, such as those observed in Figure 5, one can consider the distribution of correlation coefficients in Figure 7. We observed a marked difference in the shape of the distributions, but also an increase in the average correlation when comparing the normal karyotype with a FLT3-ITD mutation ($\mu_{FLT3-ITD}=0.6234$) versus patients without AML after chemotherapy ($\mu_{Recovery}=0.1976$) versus samples from healthy donors ($\mu_{Normal}=0.1978$). We also note the similarity in the shape of distributions between patients without AML after chemotherapy and samples from healthy donors.

## 4. Discussion

Our consideration provides an approach for both testing statistical differences between collections of correlation coefficients and detecting high-dimensional events within single cell data. The $\chi^{\delta }$ statistic presented is a global measure of differences between collections of correlation coefficients and, when there is additional structure to classify the observations in the collection, a local statistic can be derived by restricting to any subcollection defined by that additional structure. The high-dimensional cytometry data considered here has multiple levels of structure, and restricting the $\chi^{\delta }$ statistic to subsets of the data allows for the detection of potentially novel and relevant interactions between quantities measured in the cytometry panel.

More concretely, here the structure provided by surface marker expression leads to a phenotypic classification of the individual cells, and so the $\chi^{\delta }$ statistic can be localized to nodules corresponding to particular cell phenotypes. Restricting the statistic to different nodules can be a powerful tool for discerning whether the global measure of differentially correlated proteins are driven by changes in a specific phenotype or driven by many phenotypes.

Localizing the statistic affords an opportunity for correlations, and changes in correlation, to be studied within more nuanced contexts that would not be possible when only computing an aggregate measure. We began with visualizing correlations atop the minimum spanning tree, a step towards visualizing multiple protein interactions as the tree nodes are most often colored proportional to the expression of one protein of interest at a time. For the pair of interest, the visual phenomena atop the trees was explored locally within each nodule. This exploration was accomplished by considering both biaxial plots of the protein expressions, allowing us to see whether correlations are present at the single-cell level, and the distributions of their correlation coefficients, allowing quantification of differences in nodule correlation.

During the derivation of the $\chi^{\delta }$ statistic, we note that independence between the nodes cannot be confidently assumed and this is another hindrance to using the $\chi^{483}$ statistic, along with the highly variable number of cells per node discussed earlier. Regularizing the summands during the derivation and defining the $\chi^{\delta }$ statistic circumvents these challenges, but further exploration of the theoretical properties of the $\chi^{\delta }$ statistic is left for a future study. A better understanding of the statistic’s properties could help elucidate the connection between the numerical value of the statistic and visualizing the correlations atop the nodes. At this point there needs to be manual inspection of the visualizations for each statistically significant determined pair, which is a limitation of the workflow.

In the results presented in this study, for each sample, we pooled all available data at each node before correlating. There is a precedence for pooling samples for other omics datasets without a negative impact on results [30,31]. Our concern here is to demonstrate the method, so we did not explore the effect of choosing to pool samples. We recognize that pooling the samples could lead to unexpected effects like Simpson’s reversal [32] or possibly masking patient variability. If these are concerns, one would want to consider the distribution of correlations for the individual samples and compare to the pooled correlation coefficient.

The distributional comparisons shown in Figure 7 are similar to methods from Behbehani et al. [3] that characterized AML subtypes via protein aberrance, and the average correlations for these distributions help to quantify the visual differences observed in Figure 5. This is an important step when using this approach to generate hypotheses. The bimodal characteristic of the distributions for the samples from healthy donors and samples from patients without AML after chemotherapy are interesting to note, while the distribution for the normal karyotype with a FLT3-ITD mutation sample is tightly centered ($\sigma_{FLT3-ITD}=0.06287872$) about the average correlation. The standard deviations for the other distributions are each more than three times $\sigma_{FLT3-ITD}.$ Protein pairs that are differentially correlated for AML samples, but not in healthy nor recovered samples, can be important for gaining insight into drivers of the disease. Localizations can thus yield testable hypotheses on significant protein interactions.

Localization is also useful before attempting to draw insight from differential correlation. The protein pair H3Kac and CD99 was found to be differentially correlated for all sample groups globally, but when restricting to the CD34+ CD38− nodule, it was not the case for two groups: normal karyotype with FLT3-TKD mutation and patients without AML after chemotherapy. These groups are shown in Panels F or G of Figure 6. The protein expressions there, along with the the normal controls in Panel H, do not show as strong a correlation as those in Panels A–E. We note also that these groups had the smallest local $\chi^{\delta }$ contribution from CD34+ CD38− (Table 5).

The presence of the correlations at single-cell level for groups like normal karyotype with a FLT3-ITD mutation and the absence for patients without AML after chemotherapy and samples from healthy donors aligns with the averages of the correlation coefficient distributions seen in Figure 7. This leads us to conclude the statistic is detecting strong correlation and points to the necessity of considering context before reporting differential correlation results. Reporting context is important as a differential correlation for the CD34+ CD38− nodule points to an increase in correlation, but when looking at the basophils, the nodule contributions are different and there is a visual increase in negative correlation when comparing normal karyotype with a FLT3-ITD mutation versus samples from healthy donors.

Another strength of our approach is that the statistical method presented is agnostic to the clustering method used to define the subcollections of cells. Thus, we are not restricted to using SPADE before computing correlation coefficients and applying our method, but could also use VISNE [9] with binning or any other contemporary clustering approach for cytometry data.

In many of the sample groups the analysis suggested there was a strongly positive correlation in the case of H3Kac and CD99, and the CD34+ CD38− cells had a large contribution to the global statistic value here. When localizing to the CD34+ CD38− nodule, the biaxial plots (Figure 6) also suggested a strong positive correlation at the single-cell level between H3Kac and CD99 that was observed visually in the minimum spanning trees for many of the AML subtypes, as well as the lack of correlation in the samples from healthy donors and patients without AML after chemotherapy groups.

While correlation does not imply causation, localization can help to explore contexts where it might make sense to begin to consider casual relationships. The strength of the correlation between H3Kac and CD99 is interesting due to CD99’s role in diagnosis [33,34] and histone 3 being disregulated in many cancers [35]. Determining correlations in the context of specific nodules could help to generate hypotheses that can be tested experimentally to better understand histone 3 regulation in the context of leukemia, where modifications to histone acetylation are not yet well understood [36]. Moreover, the role of CD34+ CD38− in leukemia diagnosis and outcome [37,38] makes the presence of this relationship within that nodule interesting to consider.

Correlation is the most common measure of similarity in high-throughput gene expression and large-scale omics studies [5,6], so it is natural to seek a notion of testing correlation when considering entire high-dimensional cytometry datasets. Understanding differential correlations between pairs of proteins, like H3Kac and CD99, is a step towards improving the understanding of networks around proteins of interest. The localization feature helps discern exactly the correlation relationship present in different nodules.

## Figures and Tables

**Table 1:** Summary of the different subtypes of acute myeloid leukemia (AML) considered in the study by Behbehani et al. [3] and reanalyzed here. Shortnames for the different subgroups, the number of samples, and associated colors to help distinguish subgroups are given. The subtypes are compared to 14 samples from healthy donors (associated color: 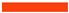) in this study.

Shortname	Full Diagnosis	# of Samples	Color
CBF	Core-binding factor AML	5	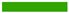
APL	t(8;21) or inv(16) karyotype	4	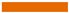
FLT3-ITD	Normal karyotype with a FLT3-ITD mutation	11	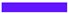
FLT3wt	Normal karyotype with wildtype FLT3	5	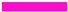
ARK	Adverse risk karyotype	7	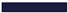
FLT3-TKD	Normal karyotype with a FLT3-TKD mutation	4	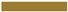
CR	Patients without AML after chemotherapy	6	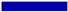

**Table 2:** Exchangeability factors to minimize the difference between the means of the summands $c_{x_{i},y_{i}}$ and 1, the mean of a $\chi_{1}^{2}.$ The $s_{0}$ value appears in the denominator of $c_{x_{i},y_{i}}$ and prevents the summand from growing too large at nodes with a large numbers of cells.

Cell Cycle $s_{0}$	Signaling $s_{0}$
0.02084245	0.01999323

**Table 3:** Summary of the number of protein pairs found to be globally differentially correlated, across all comparisons of subtype groups to normal controls, and the ad hoc false discovery rates (FDRs) at different *p*-values for each dataset. Significance levels were determined using an empirical distribution derived from comparing the normal samples under the $\chi^{\delta }$ statistic. For subsequent analysis we chose the significance level p=0.001.

Nominal *p*	Cell Cycle; n	Cell Cycle; FDR	Signaling; n	Signaling; FDR
0.100	704	0.617	841	0.517
0.050	508	0.428	703	0.309
0.010	196	0.222	423	0.103
0.005	91	0.239	311	0.070
0.001	37	0.117	150	0.029

**Table 4:** Summary of potentially novel protein pairs and the sample groups for which the pairs were found to be globally differentially correlated compared to normal control samples (denoted by an asterisk). The protein pairs detected to be significant at p=0.001 as mentioned in Table 3 were divided into the categories: positive controls, known biological relationships, and potentially novel relationships. Choosing the significance level p=0.001 led to a threshold of 1363.91. Colors convention established in Table 1 are continued here to help with distinguishability.

	CBF	APL	ITD	FLT3wt	ARK	TKD	CR
pS6; H3Kac	1251.1	779.94	1427.62 *	1496.33 *	1108.71	1196.94	795.2
pS6; CD99	845.4	587.12	1325.35	1304.58	969.55	1406.21 *	604.54
pATM; H3Kac	2054.39 *	1028.18	1959.45 *	1782.7 *	1913.49 *	1800.82 *	1404.83 *
pATM; CD99	1965.59 *	1023.14	1828.97 *	1153.73	1821.9 *	1277.02	667.2
H3Kac; pMAPK	3430.31 *	1149.44	2432.72 *	1867.3 *	2069.08 *	1069.73	1318.33
H3Kac; STAT5	921.6	1285.34	1473.73 *	1270.78	874.47	906.41	638.91
H3Kac; Ki-67	3509.44 *	3102.05 *	3507.08 *	2122.72 *	2900.5 *	2102.26 *	2806.86 *
H3Kac; CD99	4295.43 *	3471.87 *	5815.72 *	2562.15 *	3965.85 *	2719.18 *	1539.04 *
H3Kac; 4EBP1	2240.3 *	1108.39	2967.41 *	1542.32 *	2108.27 *	2113.84 *	1204.1
H3Kac; pCreb	2228.5 *	1203.15	2666.79 *	1787.46 *	2065.36 *	1426.89 *	1188.5
pMAPK; CD99	1577.94 *	788.19	1653.67 *	1394.7 *	2047.25 *	1217.4	710.59
STAT3; CD99	1442.28 *	714.58	1753.46 *	940	1232.56	924.93	593.5
STAT5; CD99	1263.89	1177.67	1407.84 *	991	1209.49	1097.8	574.29
Ki-67; CD99	1825.63 *	1701.35 *	2463.29 *	1663.45 *	1923.67 *	1400.92 *	1137.75
CD99; pErk	2157.08 *	1109.68	2012.67 *	1340.95	1332.6	1370.73 *	777.97
CD99; 4EBP1	2597.25 *	1506.13 *	3999.08 *	2898.41 *	2059.15 *	2518.81 *	937.04
CD99; pCreb	2157.67 *	2318.94 *	2805.42 *	1979.56 *	2171.93 *	1804.66 *	746.57

**Table 5:** Local $\chi^{\delta }$ statistic contributions from each immunophenotype nodule when comparing each subtype to the samples from healthy donors. The local $\chi^{\delta }$ statistic is the proportion of the global $\chi^{\delta }$ statistic that arises from any specified subset of nodes. These represent the restriction of the global $\chi^{\delta }$ statistic to nodes for the CD34+ CD38− nodule. This protein pair was found to be globally differentially correlated for each subtype, but the local contributions from the immunophenotype nodules differs for each comparison. Colors convention established in Table 1 are continued here to help with distinguishability.

	CBF	APL	ITD	FLT3wt	ARK	TKD	CR
Other	0.215	0.606	0.357	0.248	0.223	0.408	0.277
T cells	0.047	0.103	0.103	0.203	0.128	0.143	0.179
Platelets	0.008	0.012	0.055	0.084	0.077	0.011	0.073
Plasma cells	0.006	0.005	0.012	0.013	0.015	0.002	0.028
pDCs	0.026	0.012	0.012	0.006	0.022	0.009	0.030
NK cells	0.016	0.023	0.013	0.027	0.020	0.006	0.038
Mature granulocytes	0.000	0.003	0.002	0.003	0.004	0.007	0.003
Mature CD14+ Monocytes	0.063	0.016	0.057	0.085	0.037	0.116	0.052
Mature B cells	0.048	0.022	0.025	0.017	0.014	0.011	0.024
Erythroid cells	0.016	0.024	0.034	0.016	0.032	0.024	0.069
Early monocytes	0.200	0.073	0.132	0.092	0.160	0.157	0.089
Early granulocytes	0.015	0.014	0.012	0.054	0.033	0.038	0.017
Early B cells	0.010	0.002	0.001	0.006	0.004	0.010	0.041
CD34+ CD38−	0.123	0.025	0.039	0.043	0.042	0.010	0.016
CD34+	0.197	0.025	0.129	0.087	0.180	0.026	0.020
Basophils	0.010	0.033	0.014	0.015	0.008	0.021	0.044
Promyelocytes	0.000	0.001	0.001	0.002	0.000	0.001	0.000

**Figure A1:**
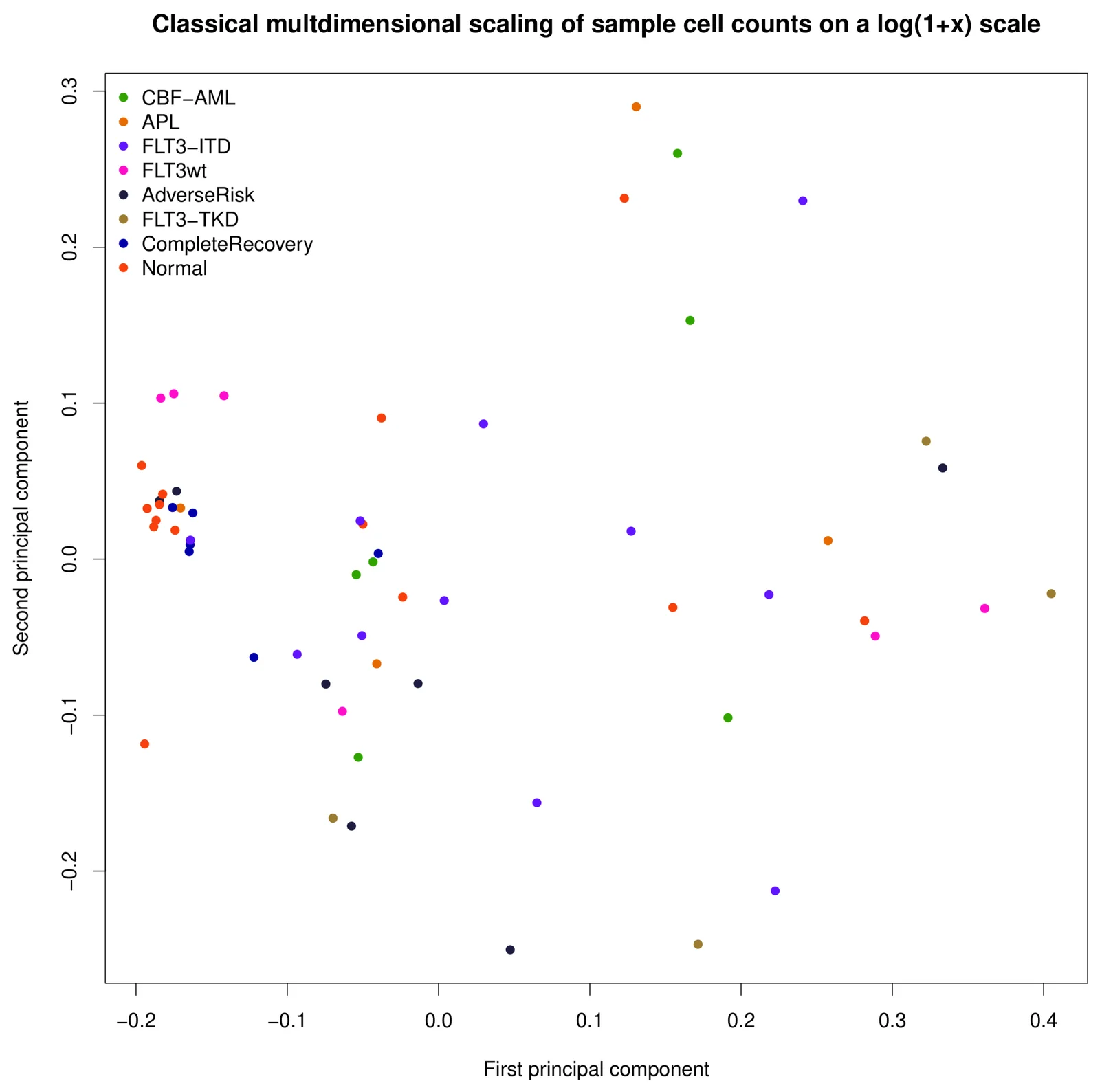
Plot of the cell counts per node for the 58 samples projected onto the first two dimensions determined by classical multidimensional scaling using the correlation distance metric. Cells in each sample were assigned to one of 483 nodes by SPADE [11] and the projection gives a two dimensional summary of the variability of how cells were allocated to the nodes. The cell counts per node are presented on the $ForLog\left(x\right)≜\log_{10}\left(1+x\right)$ scale. Samples are colored by the disease subtype. The correlation distance was chosen as the metric in the classical multidimensional scaling to avoid the first principal component being driven by cell count.

**Figure A2:**
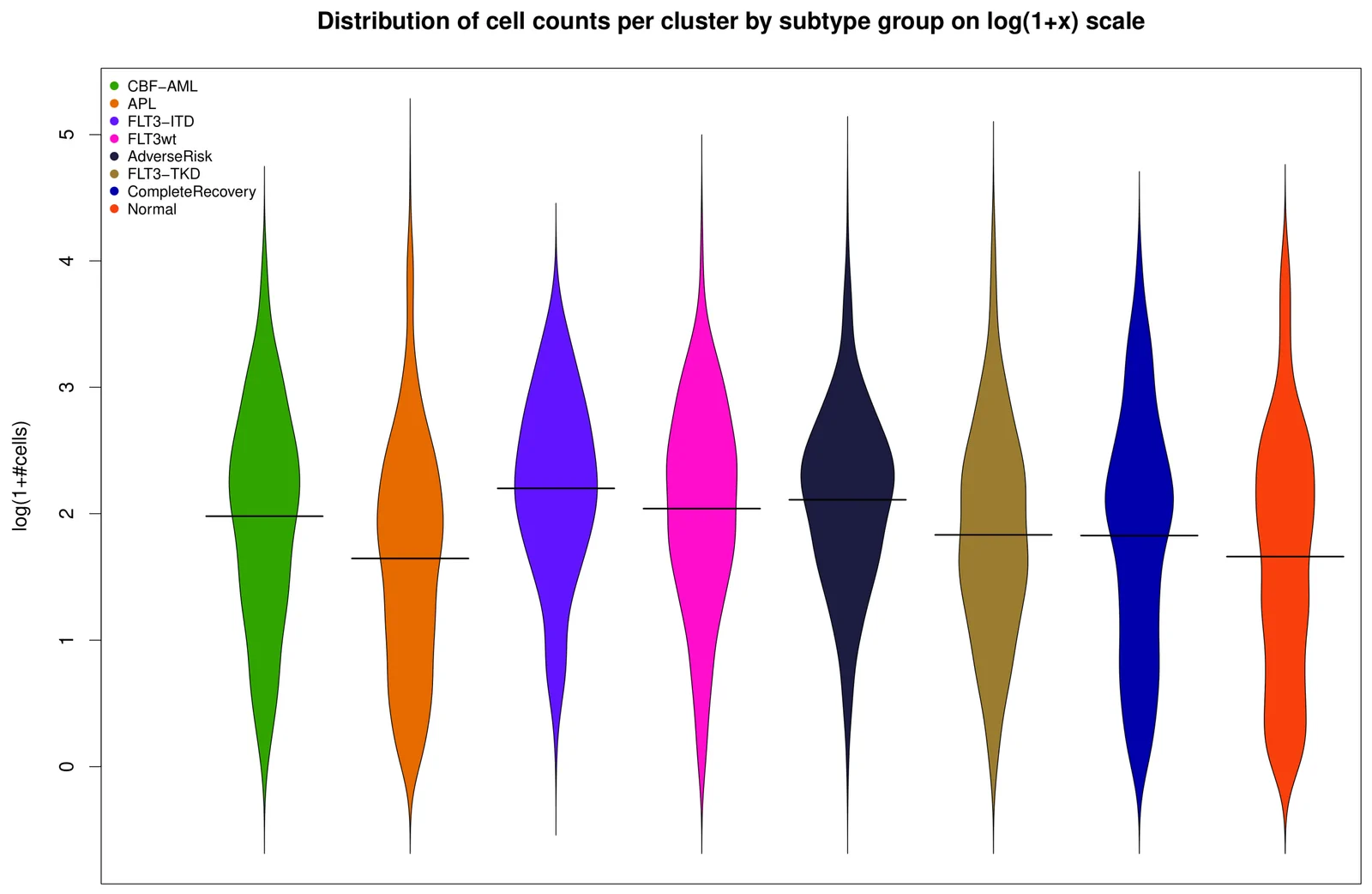
Bean plots for the distribution of cell counts per node separated and colored by subtype. The cell counts per node are presented on the $ForLog\left(x\right)≜\log_{10}\left(1+x\right)$ scale. Visually there is a clear pairwise difference of mean and variance for the subtypes. The number of cells per node was normalized by the number of cells in the group and adjusted by the average number of cells per sample. The normalization and adjustment were used to remove the effect of having a nonuniform number of samples per group on the means.

**Figure A3:**
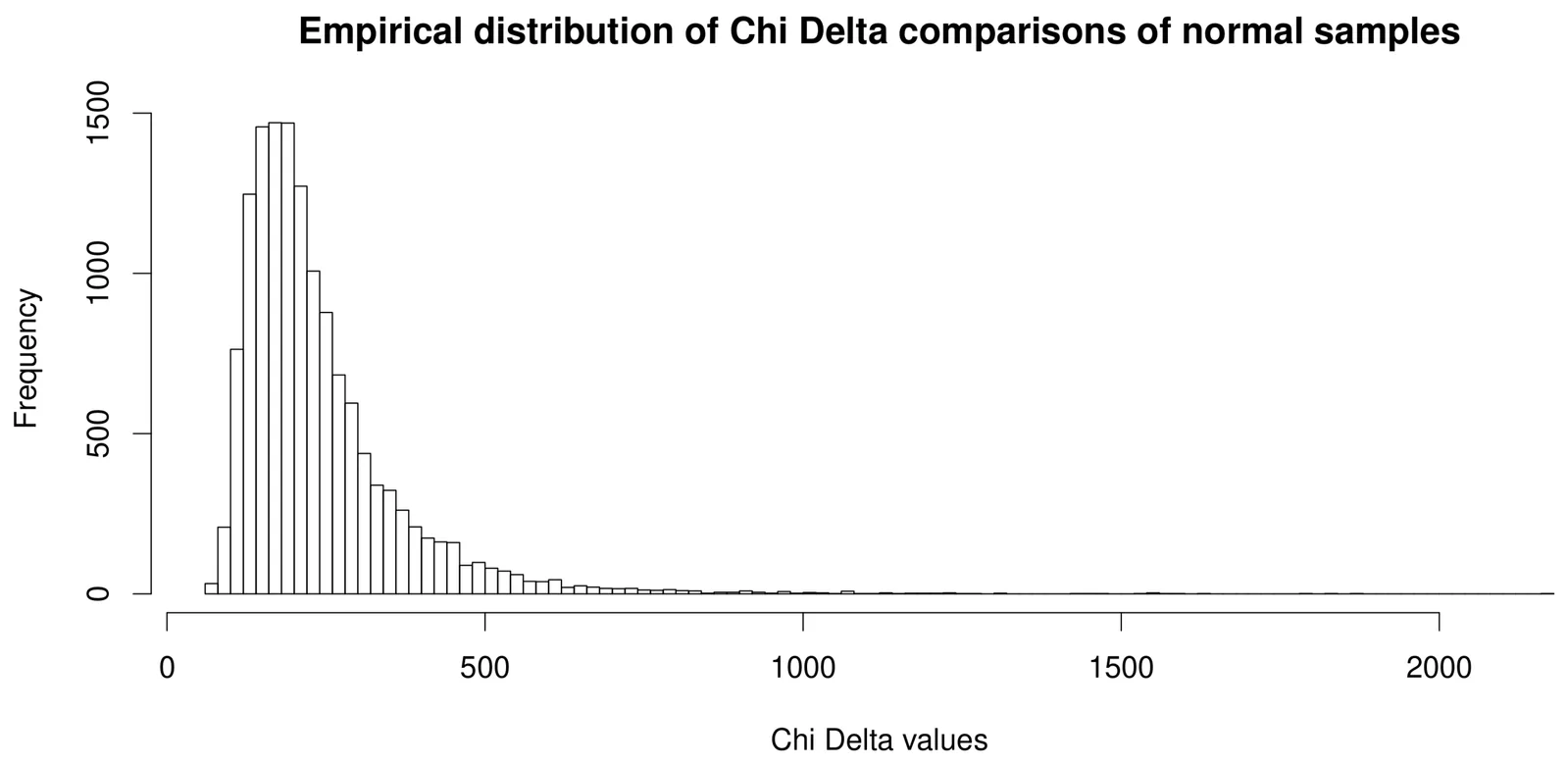
An empirically derived distribution for $\chi^{\delta }$ comparisons for the 91 possible pairs of normal samples in the case of the cell signaling panel. We show in Figure A4 that this empirical distribution of individual $\chi^{\delta }$ comparisons of samples will bound the $\chi^{\delta }$ distribution of all complementary groupings. Moreover, the empirical distribution will serve as the null distribution in subsequent statistical comparisons instead of the theoretical distribution.

**Figure A4:**
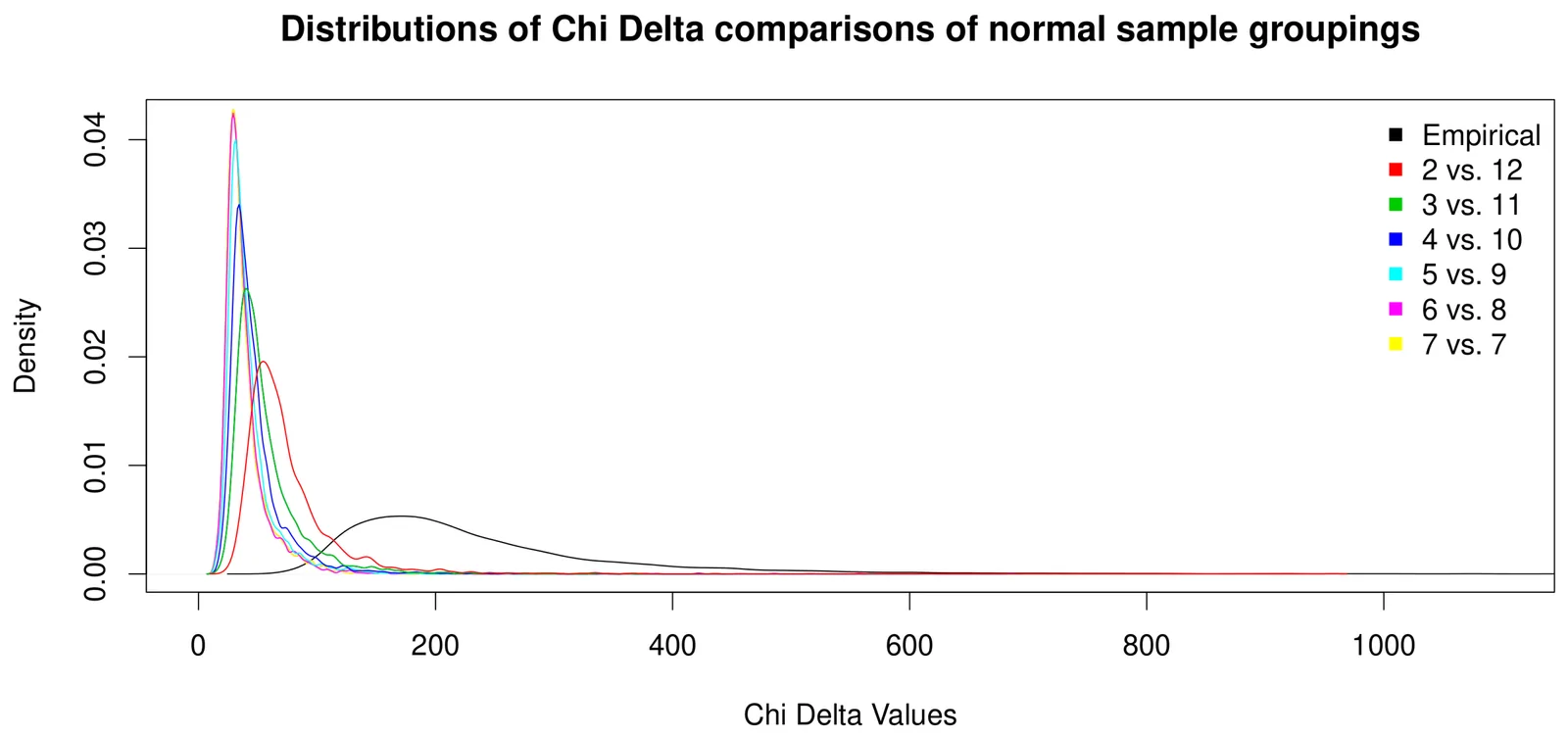
Densities of $\chi^{\delta }$ comparisons resulting from complementary groupings of normal samples. Each division of the normal samples into groups of size *m* and *n*, where m+n=14 and m,n≥2, was repeated thirty times. The discrepancy between the subgrouping distributions meant that the $\chi^{\delta }$ statistic was sensitive to the size of groups being compared, but the empirical distribution described in Figure A3 bounded each of the subgrouping distributions. This bound will allow us to circumvent a lack of independence to grouping size. Finally, the bound further ensures the empirical distribution gives a conservative measure of statistical significance.

## Data Availability

The original contributions presented in this study are included in the article. Further inquiries can be directed to the corresponding author.

## Appendix A

### Appendix A.1. Processing Clustered Data

The acute myeloid leukemia (AML) data was processed in R by four scripts. The first script set up file paths; gathered and stored information about the proteins studied (Figure 1) and AML subtypes (Table 1); and created keys on how to match subtypes with samples. This information was then stored in an R data file that is called RDA in all the subsequent scripts. The second script clustered the data in Flow Cytometry Standard (FCS) format with SPADE. SPADE outputs FCS files appended with node and density information as well as layout and Geographical Markup Language (GML) files to recreate the minimum spanning tree with the library iGraph.

The third script separates each appended FCS file into RDA files with cell information for the 483 nodes. For both the cell signaling and the cell cycle panel, the cell per node information is stored for each sample and with all samples pooled by node and samples in each subtype pooled by nodes. To account for differences in cell size within and between samples, as measured by the expression level of an irridium-based cell marker (IBC), we removed the effect of cell size to each of the features through a linear regression before computing Pearson correlations.

The fourth script estimated correlation matrices for the 18 proteins to be analyzed at each of the nodes through a Bayesian approach [25]. The estimation circumvents issues that arise when a node is empty or contains only a few cells and attempts to correlate data with built-in R functions. When there are no cells (N=0) in a given node, the method estimates the 18 × 18 correlation matrix $C_{N}$ for that node to be the identity matrix $I_{18}$. As mentioned in Section 2.4, assuming a Wishart prior distribution, the correlation matrix $C_{N}$ was estimated as follows when there are *N* cells in a node:

$$\begin{array}{cc} a_{N} & =18+\frac{N}{2},\;\;\;\beta_{N}=1+N,\\ \bar{f} & =\frac{1}{N}\sum_{n=1}^{N}f_{n},\;\;\bar{\Sigma }=\frac{1}{N}\sum_{n=1}^{N}\left(f_{n}-\bar{f}\right){\left(f_{n}-\bar{f}\right)}^{T},\\ B_{N} & =I_{18}+\frac{N}{2}\left[{\bar{\Sigma }}_{N}+\frac{1}{\beta_{N}}\bar{f}{\bar{f}}^{T}\right],\\ C_{N} & =\frac{1}{a_{N}}B_{N}. \end{array}$$

Thus, when there are many cells in a node, then the data-derived estimate overwhelms the prior $I_{18}$. Correlation matrices are estimated at every node, and stored in the folders named according to that node, for individual samples, the all-sample pool, and each subtype pool.

### Appendix A.2. Determining the Null Distribution Empirically

Despite including an exchangeability factor $s_{0}$, the variability in the number of cells per node within the mass cytometry data led to a discrepancy between the expected and the observed values of the $\chi^{\delta }$ statistic. To proceed with hypothesis testing, we chose to define an empirical distribution using the $\chi^{\delta }$ statistic to compare the fourteen normal samples. The motivating idea was that an empirical distribution defined from normal comparisons would provide a baseline for significant differences one could expect when comparing vectors of cytometric correlations and anything beyond this baseline difference could confidently be considered significant.

The 44 samples from leukemia samples were nonuniformly distributed to groups corresponding to the seven AML subtypes and we sought to compare these to the group of fourteen normal controls. We investigated the independence to grouping size to avoid introducing false positives due to differences in sizes of groups being compared to normals. Showing independence to group size would have required that the empirical distribution not change when comparing different subgroupings of normal samples. We found that the distribution of $\chi^{\delta }$ values produced from the 91 one vs. one comparisons of normal controls (Figure A3) bounded the values produced from the comparisons of *m* vs. *n* subgroupings (Figure A4), where m+n=14; we will refer to the latter as complementary subgroupings. The comparisons of the subgroupings were each rerun 30 times. Despite the lack of independence, using the distribution from one vs. one comparisons was a conservative and reasonable choice of distribution since significant findings under it would be significant in all of the distributions from the complementary subgroupings (Figure A4).

Using the empirical distribution, we considered nominal *p*-values of 0.1, 0.05, 0.01, and 0.001. When considering the nominal *p*-value of 0.001 we found the false discovery rate (FDR) value of 0.117 and 0.029 to be satisfactory for the cell cycle and cell signaling datasets, respectively. A summary of the number of significant findings and FDR values at each nominal *p*-value can be seen in Table 3. In the analysis, we choose the level p=0.001 for significance. All protein comparisons found to be significant are listed in Table A1.

**Table A1:** Summary of all differentially correlated protein pairs and the sample groups for which the pairs were found to be globally differentially correlated compared to normal control samples (denoted by an asterisk). Again, for this analysis we chose the significance level p=0.001 leading to a threshold of 1363.91. Colors convention established in Table 1 are continued here to help with distinguishability.

	CBF	APL	ITD	FLT3wt	ARK	TKD	CR
pS6; pATM	645.67	416.68	765.28	1894.12 *	1275.82	930.43	1198.51
pS6; H3Kac	1251.1	779.94	1427.62 *	1496.33 *	1108.71	1196.94	795.2
pS6; pMAPK	1183.1	579.18	1495.19 *	2308.26 *	1833.6 *	1061.19	1325.21
pS6; CD99	845.4	587.12	1325.35	1304.58	969.55	1406.21 *	604.54
pS6; BCat	672.84	374.72	656.42	1402.61 *	1123.03	650.08	751.76
pATM; H3Kac	2054.39 *	1028.18	1959.45 *	1782.7 *	1913.49 *	1800.82 *	1404.83 *
pATM; pMAPK	651.31	522.23	805.5	2807.7 *	1889.14 *	770.81	1290.26
pATM; STAT5	917.81	566.91	1381.83 *	1685.79 *	1327.41	668.56	776.68
pATM; Ki-67	1323.1	1560.38 *	3438.67 *	1602.6 *	1797.48 *	1672.1 *	947.38
pATM; CD99	1965.59 *	1023.14	1828.97 *	1153.73	1821.9 *	1277.02	667.2
pATM; pErk	570.19	415.72	897.33	1709.08 *	852.4	614.33	810.64
pATM; BCat	743.16	869.98	1117.6	1881.69 *	1538.27 *	869.03	1177.18
pATM; 4EBP1	881.82	714.74	1455.65 *	1442.22 *	1238.37	1095.35	987.61
pATM; cPARP	3976.55 *	856.85	9068.65 *	2183.41 *	3191.53 *	2643.84 *	1495.73 *
pATM; pCreb	1177.89	639.75	2004.24 *	1940.06 *	2147.14 *	1052.54	1159.37
H3Kac; pMAPK	3430.31 *	1149.44	2432.72 *	1867.3 *	2069.08 *	1069.73	1318.33
H3Kac; STAT5	921.6	1285.34	1473.73 *	1270.78	874.47	906.41	638.91
H3Kac; Ki-67	3509.44 *	3102.05 *	3507.08 *	2122.72 *	2900.5 *	2102.26 *	2806.86 *
H3Kac; pNFkB	732.36	601.34	1565.54 *	800.16	669.19	762.44	489.85
H3Kac; CD99	4295.43 *	3471.87 *	5815.72 *	2562.15 *	3965.85 *	2719.18 *	1539.04 *
H3Kac; BCat	1380.95 *	850.65	1509.89 *	1082.59	1523.85 *	903.64	910.95
H3Kac; 4EBP1	2240.3 *	1108.39	2967.41 *	1542.32 *	2108.27 *	2113.84 *	1204.1
H3Kac; pCreb	2228.5 *	1203.15	2666.79 *	1787.46 *	2065.36 *	1426.89 *	1188.5
H3Kac; DNA-2	2514.7 *	6691.73 *	6675.38 *	1505.66 *	3616.42 *	3596.51 *	2081.54 *
pMAPK; STAT5	657.21	453.7	876.09	1464.28 *	1154.04	693.33	674.34
pMAPK; Ki-67	1943.13 *	889.86	1246.52	1456.85 *	1426.95 *	1090.35	1259.36
pMAPK; CD99	1577.94 *	788.19	1653.67 *	1394.7 *	2047.25 *	1217.4	710.59
pMAPK; pErk	1516.59 *	489.34	992.68	1310.93	1262.43	659.48	665.41
pMAPK; BCat	1525.22 *	567.8	1072.98	1747.23 *	1735.14 *	650.73	1283.43
pMAPK; pCreb	1139.21	2246.29 *	1659.95 *	1193.27	1651.2 *	595.35	1331.28
STAT3; STAT5	1038.42	537.79	1118.2	1490.5 *	1137.82	613.54	994.8
STAT3; CD99	1442.28 *	714.58	1753.46 *	940	1232.56	924.93	593.5
STAT5; CD99	1263.89	1177.67	1407.84 *	991	1209.49	1097.8	574.29
Ki-67; CD99	1825.63 *	1701.35 *	2463.29 *	1663.45 *	1923.67 *	1400.92 *	1137.75
Ki-67; BCat	1461.51 *	1227.25	1085.87	1031.2	1074.78	836.82	979.85
Ki-67; 4EBP1	1979.34 *	1151.26	2329.43 *	1312.05	1726.95 *	1161.55	1253.15
Ki-67; pCreb	1499.28 *	1157.73	1379.43 *	1404.48 *	1220.87	992.27	1805.75 *
Ki-67; DNA-2	859.3	623.13	1363.44	990.01	1364.28 *	756.82	949.35
pNFkB; CD99	1086.42	768.71	1484.55 *	740.24	631.7	865.21	418.36
pNFkB; DNA-2	353.98	371.63	1453.34 *	531.3	565.43	562.65	401.34
CD99; pErk	2157.08 *	1109.68	2012.67 *	1340.95	1332.6	1370.73 *	777.97
CD99; BCat	1281.84	1213.7	1467.39 *	1581.04 *	1328.37	1087.48	724.18
CD99; 4EBP1	2597.25 *	1506.13 *	3999.08 *	2898.41 *	2059.15 *	2518.81 *	937.04
CD99; pCreb	2157.67 *	2318.94 *	2805.42 *	1979.56 *	2171.93 *	1804.66 *	746.57
CD99; DNA-2	4239.13 *	4756.04 *	10688.74 *	2428.28 *	4733.15 *	5308.94 *	1013.66
pErk; 4EBP1	2041.64 *	496.06	1369.61 *	774.02	770.01	694.08	719.48
pErk; pCreb	1903.38 *	533.09	1346.56	751.97	1361.34	724.86	653.84
pErk; DNA-2	696.79	1261.16	2030.15 *	574.13	781.85	928.34	362.67
4EBP1; pCreb	1290.7	741.91	1367.82 *	1063.22	1092.95	905.6	857.43
cPARP; pCreb	1218.67	375.74	1787.29 *	619.31	800.14	863.7	341.84
pCreb; DNA-2	598.1	677.68	1553.73 *	683.62	790.33	532.66	400.22

## References

[1] Bendall S.C., Simonds E.F., Qiu P., El-ad D.A., Krutzik P.O., Finck R., Bruggner R.V., Melamed R., Trejo A., Ornatsky O.I. (2011). Single-cell mass cytometry of differential immune and drug responses across a human hematopoietic continuum. Science.

[2] Howlander N. (2017). SEER Cancer Statistics Review 1975–2014. http://seer.cancer.gov/csr/1975_2014/.

[3] Behbehani G.K., Samusik N., Bjornson Z.B., Fantl W.J., Medeiros B.C., Nolan G.P. (2015). Mass cytometric functional profiling of acute myeloid leukemia defines cell cycle and immunophenotypic properties that correlate with known responses to therapy. Cancer Discov..

[4] Boyd A.L., Aslostovar L., Reid J., Ye W., Tanasijevic B., Porras D.P., Shapovalova Z., Almakadi M., Foley R., Leber B. (2018). Identification of chemotherapy-induced leukemic-regenerating cells reveals a transient vulnerability of human AML recurrence. Cancer Cell.

[5] Weinstein J.N., Myers T.G., O’Connor P.M., Friend S.H., Fornace A.J., Kohn K.W., Fojo T., Bates S.E., Rubinstein L.V., Anderson N.L. (1997). An information-intensive approach to the molecular pharmacology of cancer. Science.

[6] Eisen M.B., Spellman P.T., Brown P.O., Botstein D. (1998). Cluster analysis and display of genome-wide expression patterns. Proc. Natl. Acad. Sci. USA.

[7] Tenenbaum J.B., De Silva V., Langford J.C. (2000). A global geometric framework for nonlinear dimensionality reduction. Science.

[8] Roweis S.T., Saul L.K. (2000). Nonlinear dimensionality reduction by locally linear embedding. Science.

[9] Amir E., Davis K.L., Tadmor M.D., Simonds E.F., Levine J.H., Bendall S.C., Shenfeld D.K., Krishnaswamy S., Nolan G.P., Pe’er D. (2013). ViSNE enables visualization of high dimensional single-cell data and reveals phenotypic heterogeneity of leukemia. Nat. Biotechnol..

[10] Sugar I.P., Sealfon S.C. (2010). Misty Mountain clustering: Application to fast unsupervised flow cytometry gating. BMC Bioinform..

[11] Qiu P., Simonds E.F., Bendall S.C., Gibbs K.D., Bruggner R.V., Linderman M.D., Sachs K., Nolan G.P., Plevritis S.K. (2011). Extracting a cellular hierarchy from high-dimensional cytometry data with SPADE. Nat. Biotechnol..

[12] Shekhar K., Brodin P., Davis M.M., Chakraborty A.K. (2014). Automatic classification of cellular expression by nonlinear stochastic embedding (ACCENSE). Proc. Natl. Acad. Sci. USA.

[13] Platon L., Pejoski D., Gautreau G., Targat B., Le Grand R., Beignon A.S., Tchitchek N. (2018). A computational approach for phenotypic comparisons of cell populations in high-dimensional cytometry data. Methods.

[14] Lun A.T., Richard A.C., Marioni J.C. (2017). Testing for differential abundance in mass cytometry data. Nat. Methods.

[15] Weber L.M., Nowicka M., Soneson C., Robinson M.D. (2019). Diffcyt: Differential discovery in high-dimensional cytometry via high-resolution clustering. Commun. Biol..

[16] Ornatsky O., Bandura D., Baranov V., Nitz M., Winnik M.A., Tanner S. (2010). Highly multiparametric analysis by mass cytometry. J. Immunol. Methods.

[17] Lee H.K., Hsu A.K., Sajdak J., Qin J., Pavlidis P. (2004). Coexpression analysis of human genes across many microarray data sets. Genome Res..

[18] Krishnaswamy S., Spitzer M.H., Mingueneau M., Bendall S.C., Litvin O., Stone E., Pe’er D., Nolan G.P. (2014). Conditional density-based analysis of T cell signaling in single-cell data. Science.

[19] Kleftogiannis D., Gavasso S., Tislevoll B.S., van der Meer N., Motzfeldt I.K.F., Hellesøy M., Gullaksen S.E., Griessinger E., Fagerholt O., Lenartova A. (2024). Automated cell type annotation and exploration of single-cell signaling dynamics using mass cytometry. iScience.

[20] Coombes K., Brock G., Abrams Z.B., Abruzzo L.V. (2018). Polychrome: Creating and Assessing Qualitative Palettes with Many Colors. bioRxiv.

[21] Liu P., Liu S., Fang Y., Xue X., Zou J., Tseng G., Konnikova L. (2020). Recent advances in computer-assisted algorithms for cell subtype identification of cytometry data. Front. Cell Dev. Biol..

[22] Cheng L., Karkhanis P., Gokbag B., Liu Y., Li L. (2022). DGCyTOF: Deep learning with graphic cluster visualization to predict cell types of single cell mass cytometry data. PLoS Comput. Biol..

[23] Kaushik A., Dunham D., He Z., Manohar M., Desai M., Nadeau K.C., Andorf S. (2021). CyAnno: A semi-automated approach for cell type annotation of mass cytometry datasets. Bioinformatics.

[24] Kauffman S.A. (1993). The Origins of Order: Self-Organization and Selection in Evolution.

[25] Penny W. (2014). Bayesian Inference for the Multivariate Normal. https://www.fil.ion.ucl.ac.uk/~wpenny/publications/bmn.pdf.

[26] Fisher R.A. (1921). On the “probable error” of a coefficient of correlation deduced from a small sample. Metron.

[27] Bond C.F., Richardson K. (2004). Seeing the Fisher Z-transformation. Psychometrika.

[28] Tusher V.G., Tibshirani R., Chu G. (2001). Significance analysis of microarrays applied to the ionizing radiation response. Proc. Natl. Acad. Sci. USA.

[29] Astaburuaga-García R., Sell T., Mutlu S., Sieber A., Lauber K., Blüthgen N. (2024). RUCova: Removal of unwanted covariance in mass cytometry data. Bioinformatics.

[30] Kendziorski C., Irizarry R.A., Chen K.-S., Haag J.D., Gould M.N. (2005). On the utility of pooling biological samples in microarray experiments. Proc. Natl. Acad. Sci. USA.

[31] Takele Assefa A., Vandesompele J., Thas O. (2020). On the utility of RNA sample pooling to optimize cost and statistical power in RNA sequencing experiments. BMC Genom..

[32] Simpson E.H. (1951). The interpretation of interaction in contingency tables. J. R. Stat. Soc. Ser. B (Methodological).

[33] Dworzak M., Fröschl G., Printz D., De Zen L., Gaipa G., Ratei R., Basso G., Biondi A., Ludwig W.-D., Gadner H. (2004). CD99 expression in T-lineage ALL: Implications for flow cytometric detection of minimal residual disease. Leukemia.

[34] Chung S.S., Eng W.S., Hu W., Khalaj M., Garrett-Bakelman F.E., Tavakkoli M., Levine R.L., Carroll M., Klimek V.M., Melnick A.M. (2017). CD99 is a therapeutic target on disease stem cells in myeloid malignancies. Sci. Transl. Med..

[35] Audia J.E., Campbell R.M. (2016). Histone modifications and cancer. Cold Spring Harb. Perspect. Biol..

[36] Agrawal-Singh S., Isken F., Agelopoulos K., Klein H.U., Thoennissen N.H., Koehler G., Hascher A., Bäumer N., Berdel W.E., Thiede C. (2011). Genome wide analysis of histone H3 acetylation patterns in AML identifies PRDX2 as an epigenetically silenced tumor suppressor gene. Blood.

[37] Hanekamp D., Cloos J., Schuurhuis G.J. (2017). Leukemic stem cells: Identification and clinical application. Int. J. Hematol..

[38] Zeijlemaker W., Grob T., Meijer R., Hanekamp D., Kelder A., Carbaat-Ham J.C., Oussoren-Brockhoff Y.J.M., Snel A.N., Veldhuizen D., Scholten W.J. (2019). CD34^+^ CD38^−^ leukemic stem cell frequency to predict outcome in acute myeloid leukemia. Leukemia.

